# Angiotensin-converting enzyme gene polymorphism in hypertensive rural population of Haryana, India

**DOI:** 10.4103/0974-2700.55323

**Published:** 2009

**Authors:** Sumeet Gupta, Bimal K Agrawal, Rajesh K Goel, Prabodh K Sehajpal

**Affiliations:** 1 Department of Pharmacology, M. M. College of Pharmacy, M. M. University, Mullana, Haryana, India; 2 Department of Medicine, M. M. Institute of Medical Sciences, M. M. University, Mullana, Haryana, India; 3 Department of Pharmacology, Punjabi university, Patiala, Punjab, India; 4 Department of Molecular Biology and Biochemistry, Guru Nanak Dev University, Amritstar, Punjab, India

**Keywords:** Angiotensin-I converting enzyme, insertion/deletion polymorphism, essential hypertension, North Indian population

## Abstract

**Background:**

Essential hypertension is a complex genetic disorder influenced by diverse environmental factors. Of the various physiological pathways affecting the homeostasis of blood pressure, the renin-angiotensin system (RAS) is known to play a critical role. Angiotensin-I converting enzyme (ACE) is a significant component of RAS and an insertion/deletion (I/D) polymorphism in its gene has been implicated in predisposition to hypertension.

**Objective:**

The present study is aimed to determine the association, if any, of ACE I/D polymorphism with essential hypertension in a rural population of Haryana, India.

**Materials and Methods:**

The blood samples were collected from the patients visiting M. M. Institute of Medical Sciences, Mullana, Haryana. DNA from the patients (106) and control (110) specimens were isolated, amplified by PCR and analyzed employing agarose gel electrophoresis.

**Results:**

There was no significant difference in the distribution of DD, II and I/D genotypes of ACE polymorphism in essential hypertensive patients (28.8, 25.5, and 46.2%) and their ethnically matched normal control (24.5, 30, and 45.5), respectively. The two groups also presented with very similar allelic frequencies and were also found to be in Hardy-Weinberg equilibrium.

**Conclusions:**

The present study demonstrates that ACE I/D polymorphism is not a risk factor for essential hypertension in the hitherto unstudied rural population of Haryana.

## INTRODUCTION

Cardiovascular diseases are becoming a major health burden in developing countries. About 2.6 million Indian people are estimated to die due to coronary heart disease (CHD) alone by the year 2020.[1] Hypertension is one of the important risk factor for the development of CHD. It is a multifactorial and polygenic disorder in which the interaction between several candidate genes and environmental factors play a role. The renin angiotensin system (RAS) is an important regulatory mechanism for maintaining normal blood pressure, fluid and electrolyte balance and its encoding components have been proposed as independent factors for hypertension and other cardiovascular diseases.[2,3]

Angiotensin-I-converting enzyme (ACE) is a zinc metallopeptidase widely distributed on the surface of endothelial and epithelial cells and participates in producing arteriolar constriction and a rise in systolic and diastolic blood pressure. The ACE is encoded by a 21 kb gene that consists of 26 exons and is located on chromosome 17 and contains a polymorphism in the form of either insertion (I) or deletion (D) of a 287 base pair Alu repetitive sequence in intron 16.[4] This polymorphism is shown to be associated with the interpersonal variability and individuals carrying the deletion allele are associated with increased plasma ACE levels. Earlier studies have shown association between this polymorphism and several cardiovascular diseases like myocardial infarction,[5] left ventricular hypertrophy,[6] cardiomyopathy,[2] and hypertension.[7,8] Studies have been carried out on the association between the ACE I/D polymorphism and hypertension in various populations and both positive and negative association have been reported.[9–12] The present study is the first report investigating the role of this important polymorphism in a rural population of Haryana, North India.

## MATERIALS AND METHODS

### Study population

In the present investigation, the blood samples of 106 essential hypertensive patients and 110 samples of age and sex matched normal, healthy individuals as control group were collected with informed consent from M. M. Institute of Medical, Sciences, Mullana, Haryana. It is prudent to mention that it is the only mutlifacility hospital in Mullana and caters to a rural region within a radius of around 30 km. Patients were initially not on any medication and subsequently, consented for regular check up and treatment. Their follow up is up to date. The blood samples were collected in tubes containing EDTA as an anticoagulant. The samples were transported on ice to the laboratory and were processed on the same day. The isolated DNA samples were stored at −20°C till further analysis.

Various parameters like age, sex, BMI, blood pressure, and dietary patterns were recorded in a questionnaire. Blood pressure (supine) was measured after the subject had rested at least 15 minutes with the help of mercury sphygmomanometer and stethoscope by ausculatory method.[13] The recordings were done at least three times on different days. The hypertension status of the study sample was assessed using standard criteria formulated by Joint National Committee VII.[14] Unrelated subjects living in the same rural background and without any history of hypertension, diabetes and other immunosuppressive conditions were enrolled as control subjects.

### Genotyping angiotensin-I converting enzyme insertion/deletion polymorphism

DNA samples were isolated from peripheral blood lymphocytes by the standard modified inorganic method as described by Miller *et al.*[15] and quantified following standard spectrophotometric analysis. The ACE I/D polymorphism were detected by the polymerase chain reaction using the primers flanking a 287 bp insertion sequence.[4] The optimized reaction conditions consisted of 40 ng of genomic DNA in a reaction volume of 30 μl contains 0.16 μM of each primer, 30 μM of each dNTP, 10 mM Tris-HCL (pH-9.0), 1.5 mM MgCl_2_, 50 mM KCl, 0.01% gelatin, and 0.3 U of Taq DNA polymerase (Bangalore Genei, Bangalore). Amplification was carried out for 35 cycles, each cycle consisting of denaturation at 94°C for 30 s, annealing at 58°C for 20 s, extension at 72°C for 20 s and finally a 3 m extension at 72°C. The PCR products were resolved in 2% agarose gel and visualized following ethidium bromide staining. All samples, identified as DD after initial amplification, were reconfirmed with an insertion-specific primer pair:

Forward primer: 5’-GCCACTACGCCCGGCTAAT-3’;

Reverse primer: 5’-GATGTGGCCATCACATTCGTCAGAT-3’).

The reaction conditions and amplification parameters for this confirmatory reaction were the same as stated above. Known controls of each genotype were amplified with each set of samples for the ACE I/D polymorphism.

### Statistical analysis

Data analysis was done with the help of an SPSS version 7.5. Continous variables are expressed as means ± SD. Intergroup comparisons are made using students *†* test. Allele frequencies were calculated from genotype frequencies and were compared using chi-squared (χ^2^) statistics. *P* value < 0.05 was considered statistically significant.

## RESULTS

The clinical details of the hypertensive and control subjects are presented in Table 1. The mean systolic blood pressure (SBP) and diastolic blood pressure (DBP) were significantly higher in the hypertensive subjects than in the control subjects. Interestingly, the family history of hypertension and BMI among the patients was also statistically significant as compared to the normal controls. The incidence of individuals with history of smoking and alcohol consumption was higher among the hypertensive patients as compared to the controls, however the differences were statistically nonsignificant (*P* >0.05).

**Table 1:** Demographic characteristics of subjects

	Patients (%)	Control (%)	*P* value
Number	106	110	
Sex (Male:Female)	63:43	67:43	
Age	53.90 ± 13.90	51.96 ± 16.78	0.3568
Body mass index (Kg/m^3^)	27.47 ± 3.422	23.38 ± 3.782	0.001
Systolic blood pressure (mm Hg)	142.95 ± 5.87	117.69 ± 3.620	< 0.0001
Diastolic blood pressure (mm Hg)	92.44 ± 4.600	75.63 ± 4.492	<0.0001
Alcoholic (regular use)	14 (13.2)	8 (7.3)	0.1798
Smoking	7 (6.6)	4 (3.6)	0.3677
Family history	10 (9.4)	3 (2.7)	0.0383

DATA EXPRESSED AS MEAN+SD

Table 2 shows the data pertaining to all the genotypes and the allele distribution in hypertensive patients and normal healthy controls. Both the groups were in the Hardy-Weinberg equilibrium. The frequency of I/D heterozygote as compared to homozygote was higher in both the patients and control group. It was observed that the DD genotype was slightly higher than II genotype in patients as compared to control. The I/D genotypes was 46.2% in hypertensive patients while it was 45.5% in controls. However, the differences were statistically not significant. The frequency of the D allele was only marginally higher in essential hypertensive patients as compared to the normal controls.

**Table 2:** Distribution of the genotype and allele frequencies of ACE I/D polymorphism

Population (*n*)	Genotype frequencies (%)			Allele frequencies	
		
	DD	ID	II	D allele	I allele
Hypertensive (106)	30 (28.3)	49 (46.2)	27 (25.5)	0.514	0.486
Normal controls (110)	27 (24.5)	50 (45.5)	33 (30.0)	0.473	0.527

χ^2^ BASED ON ALLELE FREQUENCY [(DF)=1] (HYPERTENSIVE VS CONTROLS) = 0.694; *P* = 0.706

**Figure 1:**
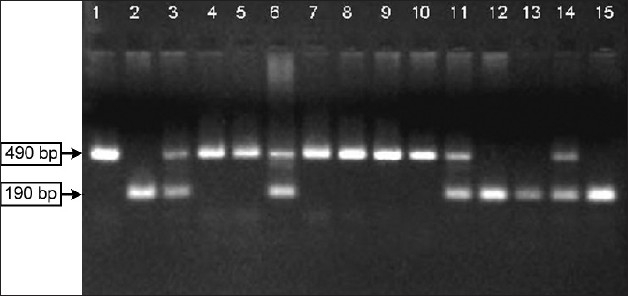
Agarose gel electrophoresis of PCR products showing the amplification for ACE I/D polymorphism. The II genotype was identified by the presence of a single 490 bp product, DD genotype was identified by the presence of a single 190 bp product and ID genotype amplified both 490 and 190bp products. The amplified products in lanes 1 and 2 are DNA sequenced PCR products of known controls of II and DD genotype, respectively

Figure 1 depicts a representative agarose gel of various genotypes of the ACE I/D polymorphism in the studied samples. Known DNA samples from II and DD subjects were amplified as controls and yielded expected products of 490 and 190bp, respectively [Figure 1, lanes 1,2]. Sample showing PCR amplified product for both the alleles were labeled as ID genotype [Figure 1, lanes 3, 6, 11, 14].

## DISCUSSION

Incidence of hypertension is increasing alarmingly in various populations of India and other developing nations.[16–18] It is universally accepted that systemic hypertension is a distinct risk factor for various cardiovascular emergencies, particularly left ventricular failure, myocardial infarction, and stroke. The present study is the first report investigating the association of ACE I/D polymorphism with hypertension in a rural population of Haryana. The strength of present study lies in extensive door-to-door preliminary investigation to identify essential hypertensive patients in villages surrounding the MM University, Ambala, Haryana, over a three-year (2003-2006) period. Preliminary survey identified a total of 2,295 hypertensive subjects, of which 930 were essential hypertensive and were not on any medication. These essential hypertensive patients were persuaded to visit Medical College at Mullana, Haryana for further investigations and 106 (∼11%) of them consented to be part of this study. The consent rate might appear low but one has to keep in mind that these subjects belong to rural area, where rate of illiteracy is very high, and despite three blood pressure measurements on different days indicating elevated levels a few of them refused to accept that they have hypertension.

Pooling of epidemiology studies show that hypertension is present in 25% urban and 10% rural subjects in India.[19] Clearly suggesting that urban conditions somehow increase the prevalence of this disease. Therefore, another forte of the study lies in the fact that patients living in the rural area were studied in the same conditions thus minimizing the influence of urban environment on the disease condition. Family history and body mass index in the hypertensive patients shows statistically significant difference from the control population [Table 1] and it does suggest that genetic factors and body mass index do influence the ability to develop this disease in the studied rural population of Haryana. These observations are in line with earlier report providing evidence that heritable factors in combination with a number of recognized environmental risk factors are important determinants of the pathogenesis and natural history of essential hypertension.[20]

It is important to ascertain gene(s) that are involved in hypertension. This would help in identifying individuals at an increased risk of developing this disease and to initiate appropriate actions in them to avoid development or delay the onset of disease. Genome wide scan and candidate gene approach are two strategies used in dissecting complex genetic diseases.[21] The former, links specific chromosomal region with inheritance of the disease, is technically cumbersome and requires sophisticated infrastructure. The candidate gene approach targets selected gene with defined polymorphism(s) for their association with the disease. The polymorphism could exist as single nucleotide change, insertion/deletion of nucleotide sequence or repetitive DNA elements. A gene and its selected polymorphism preferably should have the following features to make them a candidate target:

- The gene product must be functionally relevant to hypertension
- Polymorphism within the gene must alter its function
- Hypertension needs to link to the chromosomal region harboring the candidate gene.

Available studies demonstrate that the ACE I/D polymorphism fulfills above mentioned criterions in the context of hypertension[7,22–24] and was therefore investigated in the present study.

The frequencies of different genotypes were found to be similar in patient and the control population [Table 2]. The frequencies of both the alleles (I/D) are quite high in the control and cases, thus obviating the possibility that the frequency of the rare allele is a cause for concern in the studied sample. Lack of association between ACE I/D polymorphism and essential hypertension have been reported by investigators in Indian and other populations of the world.[25–28,40] Ethnic background is known to influence the ACE I/D polymorphism globally.[29,30] A significant association of the ACE high producing D allele with hypertension in African, Americans, Chinese, and Japanese populations have already been reported.[8,22–24] However, two studies from Australia[31] and Pakistan[32] recorded the association of I allele with hypertension. The association of I allele with hypertension in Pakistan population was attributed to limited number of individuals studied[32] and to the presence of high levels of inbreeding.[25]

The frequency of D allele of ACE I/D polymorphism in different hypertensive populations of India varied within 0.522 to 0.409 [Table 3]. The highest frequency was reported in a Sikh group from Punjab that also showed an association between the D allele and the hypertension. Similar observations have also been made on populations from other states of India.[30] The frequency of D allele in the studied patient and control populations were well within the reported range for the North Indian populations [Table 3]. Contrary to the earlier findings, no association between D allele and essential hypertension was observed in the rural population of Haryana. We believe the number of patients studied in other Indian populations showing positive associations with D allele [Table 3] were very small to allow any meaningful conclusion.

**Table 3:** The genotypic distribution and allele frequencies of the ACE I/D polymorphism in essential hypertension in different population of the world

Population studied (*n*)	Genotype distribution			Frequencies		Reference
			
	DD	ID	II	D allele	I allele
North Indian (106)	30	49	27	0.514	0.496	Present study
European						
Italian (86)	31	41	14	0.60	0.40	Teresa *et al*.[35]
Kyrgyz (180)	20	91	69	0.36	0.64	Polupanov *et al*.[36]
Dutch (257)	80	138	39	0.58	0.42	Schut *et al*.[37]
Turkish (109)	49	59	01	0.73	0.27	Agachan *et al*.[38]
Solvenian (413)	132	199	82	0.57	0.43	Galvnik *et al*.[39]
German (621)	167	309	145	0.517	0.483	Mondry *et al*.[40]
Asians							
Japanese (87)	17	26	44	0.35	0.65	Ishigami *et al*.[41]
Chinese (189)	18	77	94	0.70	0.30	Lee *et al*.[42]
Indians						
Punjabi, Punjab (100)	17	58	25	0.46	0.54	Randhawa *et al*.[23]
Sikh, Punjab (45)	12	21	11	0.522	0.477	Pasha *et al*.[28]
Jat, Haryana (30)	03	21	06	0.45	0.55	Pasha *et al*.[28]
Dogra, HP (52)	12	21	19	0.432	0.567	Pasha *et al*.[28]
Kumaonese (Uttranchal) (33)	07	13	13	0.409	0.590	Pasha *et al*.[28]
Assamese (52) (Assam)	08	28	16	0.424	0.576	Pasha *et al*.[28]

Identifying association between a gene and a complex genetic disease is difficult. One possible reason for this is the involvement of a large number of genes in the etiology of essential hypertension. Furthermore, these genes may interact with each other in different combinations to give rise to a similar disease phenotype. The magnitude of this problem makes the frequency of any polymorphism contributing to a disease phenotype marginally higher in disease group compared with unaffected controls.[33] Linkage analysis has limited power to detect such small effects[34] and case control studies with matched controls from the same population had greater probability of detecting such minute effects.[21] The inability to find association between ACE I/D polymorphism and hypertension in the present study strongly point out that ACE gene is not playing a predominant role in the pathophysiology of this disease in our population and is not a good predictor of susceptibility to hypertension. Similar observations have also been made in a Meta analysis studying the role of genetic polymorphisms in hypertension.[40] Since hypertension is a complex genetic disorder, it is assumed that there could be other genetic and environmental factors that interact and influence the development of this disease.

The results of the present study has triggered two very valid questions, i) what is the effect of different ACE I/D genotypes on the progression of the disease? and ii) are different drug regimens required for individuals with different ACE I/D polymorphism? Interestingly, our preliminary observations do suggest that hypertensive patients with DD phenotypes require higher doses of ACE inhibitor in their drug regimen as compared to their II counterparts (data not shown). The information will be of immense use in tailoring individualized therapy to hypertensive patients based on the ACE I/D genotypes.

## CONCLUSION

Our study suggests that the ACE I/D polymorphism is not a risk factor for the development of essential hypertension in the studied rural population from Haryana.
